# Fast Microbiology: A Pilot Study Comparing Culture-Independent Digital PCR and Blood Culture for Pathogen Detection in Sepsis

**DOI:** 10.3390/ijms27156849

**Published:** 2026-07-30

**Authors:** Maria Vittoria Ristori, Tiziana Marfoli, Marina De Cesaris, Sara Elsa Aita, Raffaella Rosy Vescio, Francesco Travaglino, Silvia Spoto, Raffaele Antonelli Incalzi, Silvia Angeletti

**Affiliations:** 1 National PhD Program in One Health Approaches to Infectious Diseases and Life Science Research, Department of Public Health, Experimental and Forensic Medicine, University of Pavia, 27100 Pavia, Italy; mariavittoria.ristori01@universitadipavia.it; 2 Operative Research Unit of Laboratory, Fondazione Policlinico Universitario Campus Bio-Medico, Via Alvaro del Portillo, 200, 00128 Rome, Italy; t.marfoli@policlinicocampus.it (T.M.); m.decesaris@policlinicocampus.it (M.D.C.); raffaella.vescio@policlinicocampus.it (R.R.V.); 3 Research Unit of Clinical Laboratory Science, Department of Medicine and Surgery, Università Campus Bio-Medico di Roma, Via Alvaro del Portillo, 21, 00128 Rome, Italy; 4 Operative Research Unit of General Surgery, Fondazione Policlinico Universitario Campus Bio-Medico, 00128 Rome, Italy; s.aita@policlinicocampus.it; 5 Emergency Medicine Department, Semi-Intensive Respiratory Covid Unit—Campus Covid Center, Fondazione Policlinico Universitario Campus Bio-Medico, Via Alvaro del Portillo, 200, 00128 Rome, Italy; f.travaglino@policlinicocampus.it; 6 Diagnostic and Therapeutic Medicine Department, Fondazione Policlinico Universitario Campus Bio-Medico, Via Alvaro del Portillo, 200, 00128 Rome, Italy; s.spoto@policlinicocampus.it; 7 Operative Research Unit of Internal Medicine, Fondazione Policlinico Universitario Campus Bio-Medico, Via Alvaro del Portillo, 200, 00128 Rome, Italy; r.antonelli@policlinicocampus.it; 8 Research Unit of Internal Medicine, Department of Medicine and Surgery, Università Campus Bio-Medico di Roma, Via Alvaro del Portillo, 21, 00128 Rome, Italy

**Keywords:** bloodstream infections, sepsis, digital PCR, dPCR, blood culture, rapid diagnostics

## Abstract

Bloodstream infections (BSIs) represent a major clinical challenge, particularly in patients with suspected sepsis, where rapid pathogen identification is critical for timely and appropriate antimicrobial therapy. Blood culture (BC) remains the reference diagnostic method but is limited by a long turnaround time and reduced sensitivity, especially in patients receiving prior antibiotics. This pilot study aimed to evaluate the diagnostic performance and clinical added value of culture-independent digital PCR (dPCR) performed directly on whole blood compared with paired BC. A total of 37 samples from 27 patients with suspected BSI admitted to a tertiary-care hospital were analyzed. dPCR demonstrated the ability to detect a broader spectrum of pathogens compared with BC (34 vs. 28 species), with improved identification of fastidious organisms and fungi, particularly *Candida* spp. Diagnostic performance analysis showed a sensitivity of 75.0% and a specificity of 53.8%, with a positive predictive value of 75.0% and a negative predictive value of 53.8%. Likelihood ratios indicated moderate rule-in capability (Likelihood ration positive 1.62) but limited rule-out performance (Likelihood ratio negative 0.46). Importantly, concordance analysis integrating microbiological and clinical data revealed that a substantial proportion of discordant dPCR-positive results were clinically plausible, suggesting detection of infections not captured by BC, particularly in polymicrobial settings, under antibiotic pressure, or in fungal infections. Longitudinal observations further highlighted the potential of dPCR for monitoring pathogen dynamics and treatment response. Overall, dPCR provides rapid and complementary diagnostic information, enabling earlier pathogen detection, identification of antimicrobial resistance markers, and improved characterization of complex infections. While not suitable as a standalone test due to limited negative predictive value, its integration with conventional microbiology may enhance diagnostic accuracy and support antimicrobial stewardship in the management of BSIs.

## 1. Introduction

Infectious diseases continue to represent a substantial global health burden, with bloodstream infections (BSIs) standing among the most severe and life-threatening clinical manifestations [1]. The presence of viable pathogens within the bloodstream reflects a failure of host containment mechanisms and frequently triggers a dysregulated systemic inflammatory response that may rapidly evolve into sepsis, septic shock, and multi-organ dysfunction [2,3,4,5]. The clinical impact of BSIs is particularly pronounced in critically ill and immunocompromised patients, where delayed or inappropriate antimicrobial therapy is strongly associated with poor outcomes and increased mortality. In this context, rapid and reliable pathogen identification is a cornerstone of effective clinical management and antimicrobial stewardship [6].

Blood culture (BC) remains the reference diagnostic method for BSI due to its ability to provide pathogen isolation and antimicrobial susceptibility testing [7]. Nevertheless, its clinical utility is frequently compromised by limited sensitivity and prolonged turnaround time [8,9,10]. Several factors contribute to suboptimal BC performance, including prior antimicrobial exposure, low circulating pathogen burden, pathogens requiring specific and stringent culture conditions, blood culture contamination and variability in pre-analytical and laboratory practices [11,12,13,14]. As a result, clinically relevant infections may remain microbiologically undocumented, particularly during early disease stages or in patients already receiving empirical therapy [8,10,13,15]. These limitations highlight a critical unmet need for diagnostic strategies capable of providing faster and more sensitive pathogen detection directly from blood samples [8,15].

Molecular diagnostic approaches have emerged as important adjuncts to conventional microbiology, enabling detection of microbial nucleic acids independently of organism viability [8,11,15,16]. PCR-based assays have demonstrated improved sensitivity for pathogen detection in low-abundance settings; however, conventional quantitative PCR techniques may be affected by amplification efficiency variability, the requirement for calibration curves, and limited precision at very low target concentrations [17,18,19,20]. Digital PCR (dPCR) represents a technological advancement that addresses several of these challenges by partitioning the sample into thousands of individual reactions, allowing absolute quantification of nucleic acid targets based on Poisson statistics [20,21,22]. This approach provides enhanced sensitivity, improved tolerance to inhibitors, and robust quantification without reliance on external standards [17,20,21,22,23,24,25].

The ability of dPCR to detect and quantify trace levels of pathogen DNA in whole blood introduces clinically relevant opportunities extending beyond early diagnosis [26,27,28,29,30,31]. Quantitative molecular detection may offer insight into pathogen burden dynamics, treatment response, and microbiological persistence, particularly in scenarios where blood cultures remain negative due to antimicrobial suppression or low microbial viability [26,28,29,30]. Although dPCR has been successfully applied in multiple clinical domains—including oncology, transplantation monitoring, and prenatal diagnostics—its role in the routine evaluation of suspected BSIs remains incompletely defined [21,26,27,29,30,31,32]. Evidence describing its real-world diagnostic performance, interpretation of discordant molecular–culture results, and potential contribution to clinical decision-making across heterogeneous patient populations is still limited [26,27,29,30,31].

Considering these factors, the present study aimed to investigate the diagnostic utility of direct blood dPCR in patients with suspected BSI through comparison with paired blood cultures. Specifically, we evaluated diagnostic concordance, quantified test performance metrics, and explored the relationship between dPCR quantitative results and clinical features. By integrating microbiological and clinical data, this work seeks to clarify the potential role of dPCR as a complementary tool for rapid pathogen detection and to better define its contribution to the diagnostic pathway of bloodstream infections in real-world clinical settings.

## 2. Results

### 2.1. Patients’ Characteristics

The study population included 27 patients admitted to the Fondazione Policlinico Campus Bio-Medico di Roma between December 2025 and February 2026. Patients were recruited from multiple hospital departments, including cardiac surgery, general surgery, hematology, hepatology, geriatrics, immunohematology, internal medicine, clinical medicine, emergency medicine, medical pathology, the emergency room, intensive care, and urology, reflecting a heterogeneous clinical setting of suspected bloodstream infections. A total of five patients underwent longitudinal sampling, allowing follow-up analysis over time. For each patient, a comprehensive dataset was collected, including demographic characteristics, clinical data, severity scores, and laboratory biomarkers (Table 1). The median age of the cohort was 69 years (IQR 56–78), with a slight male predominance (52%). Laboratory findings showed a median white blood cell (WBC) count of 5.3 × 10^3^/µL (IQR 2.7–10.5), indicating wide variability in the immune response among patients. Median procalcitonin (PCT) levels were 0.5 ng/mL (IQR 0.2–2.1), while median C-reactive protein (CRP) levels were 8.7 mg/L (IQR 2.6–11.9), consistent with an inflammatory state across the cohort. Overall, the study population represents a clinically heterogeneous group of patients with suspected sepsis or bloodstream infection, characterized by variable inflammatory and hematological profiles.

### 2.2. Pathogens Identified by dPCR and Blood Culture

A total number of pathogens detected by dPCR and BC was compared across the study cohort. Overall, dPCR identified a broader spectrum of microorganisms, detecting 34 species (28 bacteria and 6 fungi), whereas blood culture identified 28 species (26 bacteria and 2 fungi). At the genus and species level, relevant differences in detection rates were observed between the two methods (Table 2). dPCR showed higher detection rates for several pathogens, including *Enterococcus* (6 vs. 5), *Pseudomonas aeruginosa* (4 vs. 2), *Streptococcus* spp. (2 vs. 0), *Acinetobacter baumannii* (2 vs. 1), *Proteus mirabilis* (2 vs. 0), and *Klebsiella pneumoniae* (5 vs. 2). Notably, dPCR also identified a higher number of fungal pathogens, particularly *Candida* spp. (6 vs. 2). In contrast, blood culture yielded higher detection rates for coagulase-negative staphylococci (8 vs. 5), *Escherichia coli* (4 vs. 2), and *Staphylococcus aureus* (5 vs. 3). Additionally, some microorganisms were exclusively identified by blood culture, including *Corynebacterium striatum* (2 cases) and *Citrobacter freundii* (1 case), which were not detected by dPCR because they were not included in the dPCR panel. Regarding negative results, dPCR reported a higher number of negative samples compared with blood culture (12 vs. 8), suggesting potential differences in analytical sensitivity, pathogen load, or clinical context at the time of sampling. Overall, these findings indicate that dPCR enables a broader and partially complementary detection of pathogens compared with conventional blood culture, particularly for fastidious organisms and fungi, while blood culture remains relevant for specific bacterial groups. Quantitative dPCR results are reported in Supplementary Table S1.

### 2.3. Diagnostic Performance of dPCR

The diagnostic performance of dPCR was evaluated using blood culture (BC) as the reference standard (Table 3). Among the analyzed samples, dPCR correctly identified 18 true positive (TP) cases and 7 true negative (TN) cases. A total of six false negative (FN) results were observed, where dPCR failed to detect pathogens identified by BC, while six false positive (FP) cases were recorded, corresponding to pathogens detected by dPCR but not confirmed by BC. Notably, a subset of these false-positive results corresponded to clinically plausible detections, including cases with documented infection at other anatomical sites or with microbiological evidence suggesting polymicrobial or fungal involvement, highlighting the potential of dPCR to identify pathogens not captured by blood culture.

Using blood culture as the reference standard, dPCR demonstrated a sensitivity of 75.0% (95% CI, 53.3–88.8%) and a specificity of 53.8% (95% CI, 29.1–76.8%). The positive predictive value (PPV) was 75.0% (95% CI, 55.1–88.0%), whereas the negative predictive value (NPV) was 53.8% (95% CI, 29.1–76.8%). Cohen’s kappa coefficient demonstrated fair agreement between methods (κ = 0.29). Likelihood ratio analysis showed a positive likelihood ratio (LR+) of 1.62 and a negative likelihood ratio (LR−) of 0.46. As expected for this pilot study, the relatively wide confidence intervals reflect the limited sample size and should be considered when interpreting the precision of these estimates.

Overall, these findings suggest that dPCR provides a high rule-in capability, with strong agreement in positive cases, while its rule-out performance remains limited compared with blood culture. To better interpret the diagnostic performance of dPCR, results were further analyzed according to concordance with blood culture and clinical context (Table 4). Cases were categorized as concordant positive, concordant negative, or discordant, with discordant results further subdivided into clinically plausible and likely due to contamination. Overall, concordant positive cases showed strong agreement between dPCR and blood culture in identifying the same pathogen, confirming bloodstream infection. Concordant negative cases were consistent across both methods, indicating the absence of detectable infection. Notably, a substantial proportion of discordant cases was classified as clinically plausible. These included dPCR-positive results in patients with supporting microbiological evidence from other anatomical sites, polymicrobial infections, prior antibiotic exposure, or fungal involvement. In such scenarios, dPCR appeared to detect pathogens not captured by blood culture, likely due to its higher sensitivity and independence from microbial viability. Conversely, discordant cases without clinical or microbiological support were considered likely due to contamination, particularly when involving typical skin commensals or single-timepoint detections. Overall, this concordance-based approach highlights that the apparent false-positive rate of dPCR may be overestimated when using blood culture as the sole reference standard. Integration of clinical context suggests that dPCR provides additional diagnostic value by identifying clinically relevant infections that may otherwise remain undetected, particularly in complex or polymicrobial settings. In addition to its diagnostic performance, dPCR provided a substantial advantage in terms of turnaround time. The complete dPCR workflow, including DNA extraction and analysis, required approximately 3–5 h from sample processing to result availability. In contrast, blood cultures required 18–48 h to signal positivity, whereas definitive pathogen identification and antimicrobial susceptibility testing (AST) generally required 48–96 h. This shorter time-to-result may enable earlier optimization of antimicrobial therapy and support timely clinical decision-making (Supplementary Table S2).

### 2.4. Clinical Added Value of dPCR in the Study Cohort

Within the overall dataset, dPCR demonstrated a relevant clinical added value by enabling early pathogen detection, quantitative monitoring, and the identification of polymicrobial dynamics that were not always captured by blood culture. Longitudinal cases highlighted the ability of dPCR to track changes in pathogen load over time and to detect emerging or persistent infections under antibiotic pressure. These findings support the role of dPCR as a complementary diagnostic tool, particularly in complex clinical scenarios such as non-responsive infections or suspected polymicrobial sepsis. The high positive predictive value observed in the cohort further reinforces its utility as a reliable rule-in method, while its quantitative output provides actionable information for therapy monitoring and optimization.

### 2.5. Case-Based Clinical Insights

Across the study cohort, selected clinical cases highlighted the added value of dPCR in complex infectious scenarios, particularly in the identification of polymicrobial infections, monitoring of infection dynamics, and early detection of antimicrobial resistance-associated genes and fungal involvement (Table 5). In one case, a 40-year-old female patient admitted to the emergency department with suspected bloodstream infection showed initial detection of *Enterococcus faecalis* at low levels by dPCR. Despite empirical antibiotic therapy with ceftriaxone, the patient did not improve clinically. Follow-up dPCR analysis revealed a marked increase in *Enterococcus faecalis* load, indicating persistent infection, and additionally identified *Pseudomonas aeruginosa*, suggesting a polymicrobial etiology that may have contributed to therapeutic failure. Another case involved a 64-year-old female patient in whom dPCR detected *Candida* spp. in peripheral blood, with persistence over time. Microbiological data revealed prior positivity for methicillin-resistant *Staphylococcus aureus* and a pre-existing untreated urinary infection caused by *Candida albicans*. The coexistence of bacterial and fungal findings, together with temporal discrepancies between dPCR and blood culture, pointed to a complex infectious scenario potentially involving transient fungal dissemination or polymicrobial infection. A further example was a 66-year-old female patient in whom dPCR initially identified a high load of *Escherichia coli* along with *Proteus mirabilis*, consistent with a Gram-negative infection. Following antibiotic treatment with meropenem, dPCR revealed a substantial shift in the microbial profile, with a reduction in E. coli and the emergence of additional pathogens, including *Pseudomonas* spp., *Klebsiella pneumoniae*, and carbapenemase-producing organisms. This finding suggests either the selection of resistant organisms under antibiotic pressure or the unveiling of a previously unrecognized polymicrobial infection. In a 68-year-old male patient admitted to hematology, dPCR detected low levels of *Enterococcus* spp., which was subsequently confirmed as *Enterococcus faecium* (vancomycin-resistant) in a wound culture. This concordance suggests a possible link between localized infection and systemic dissemination. In contrast, the simultaneous detection of coagulase-negative staphylococci was interpreted as a likely contamination or transient presence. In an elderly frail patient (88 years old), dPCR identified both *Streptococcus* spp. and *Staphylococcus aureus*, with prior blood culture confirming *S. aureus*. Additional microbiological findings, including *Enterococcus faecium* from urine, indicated a complex and potentially polymicrobial infection involving multiple sources, consistent with the patient’s clinical vulnerability. A particularly severe case involved a 77-year-old male patient in the intensive care unit, where dPCR detected *Acinetobacter baumannii*, *Klebsiella pneumoniae*, and carbapenemase-associated markers, indicating a multidrug-resistant infection. These findings were supported by blood culture and multiple other clinical samples, which consistently demonstrated a disseminated polymicrobial infection involving several Gram-negative pathogens. In another case, an 82-year-old male patient with a documented urinary infection due to *Candida albicans* showed detection of *Candida* spp. in blood by dPCR. This finding suggests a potential link between the urinary source and transient or systemic fungal dissemination, highlighting the role of dPCR in identifying fungal involvement even in the absence of immediate blood culture confirmation. Finally, in an 85-year-old male patient, dPCR identified a high load of *Klebsiella pneumoniae* along with carbapenemase genes (NDM and OXA-48), indicating a multidrug-resistant strain, and concurrently detected *Candida* spp. Subsequent blood culture confirmed *Candida parapsilosis*, supporting the clinical relevance of the early molecular detection. This case exemplifies the ability of dPCR to simultaneously provide information on antimicrobial resistance and fungal infection. Overall, these cases illustrate how dPCR extends beyond simple pathogen detection by enabling early identification of polymicrobial infections, monitoring of infection evolution, detection of antimicrobial resistance-associated genes markers, and recognition of fungal involvement, thereby supporting more informed and timely clinical decision-making.

### 2.6. Cases of Probable Contamination

Within the study cohort, several cases were classified as probable contaminants based on microbiological patterns, a lack of concordance across samples, and clinical context.

A 55-year-old female patient admitted to the hematology unit showed negative dPCR results, while a blood culture from a central venous catheter (CVC) yielded *Staphylococcus epidermidis*. Given the well-known role of coagulase-negative staphylococci as skin commensals and the absence of molecular confirmation, this finding was interpreted as a likely contamination. Similarly, in a 74-year-old male patient admitted to the emergency department, dPCR was negative, while a blood culture identified *Staphylococcus caprae*. In the absence of supporting clinical or molecular evidence, this isolate was also considered a probable contaminant. In contrast, in another 55-year-old female patient in hematology, dPCR and blood culture demonstrated the detection of *Pseudomonas aeruginosa* across multiple time points, supporting its role as a true pathogen. However, the additional detection of *Streptococcus salivarius* in one sample of BC was considered a probable contaminant, given its typical oral origin and lack of consistent detection. In a 73-year-old female patient in hematology, dPCR results were negative, while *Escherichia coli* was detected in the blood culture at a single time point only, both from peripheral and CVC samples. The lack of persistence across multiple samples suggests a low likelihood of a true bloodstream infection, supporting its classification as a probable contamination. Finally, in a 64-year-old female patient admitted to the intensive care unit, dPCR was negative, while *Staphylococcus epidermidis* was detected at only one anatomical site. Given the absence of corroborating findings, this result was also interpreted as a probable contaminant.

## 3. Discussion

The present study evaluated 27 patients with suspected bloodstream infections admitted to the Fondazione Policlinico Campus Bio-Medico di Roma between December 2025 and February 2026. The study population represents a clinically heterogeneous group, with a median age of 69 years and variable inflammatory profiles, as evidenced by procalcitonin levels (median 0.5 ng/mL) and C-reactive protein levels (median 8.7 mg/L). This heterogeneity reflects the diagnostic complexity of bloodstream infections in the hospital setting, where patients from different departments present with varying clinical presentations and bacterial loads. The results demonstrate that dPCR identified a greater number of microbial species (34 total: 28 bacteria and 6 fungi) compared with blood culture (28 total: 26 bacteria and 2 fungi). This difference is particularly evident for several key pathogens. dPCR showed higher detection rates for *Enterococcus* (6 vs. 5), *Pseudomonas aeruginosa* (4 vs. 2), *Klebsiella pneumoniae* (5 vs. 2), and especially for *Candida* spp. (6 vs. 2). Additionally, dPCR identified pathogens not detected by blood culture, such as *Streptococcus* spp. (2 cases) and *Proteus mirabilis* (2 cases) [26,28,30]. These data are consistent with recent literature highlighting the ability of dPCR to detect fastidious pathogens and fungi with greater sensitivity than traditional blood culture [26,27,28]. A multicenter study in patients with suspected sepsis demonstrated that PCR/ESI-MS molecular methods showed 83% sensitivity for *Candida* spp. and 78% for Gram-negative bacteria, with specificities exceeding 94% [33]. The ability of dPCR to detect microbial DNA even in the presence of empirical antibiotic therapy represents a significant advantage, as blood culture may yield false-negative results in these cases [26,33,34]. Using blood culture as the reference standard, dPCR demonstrated a sensitivity of 75.0% and a specificity of 53.8%. The high positive predictive value (75.0%) indicates that a positive dPCR result corresponds with a high probability to a true infection. However, the limited negative predictive value (53.8%) indicates a limited ability to confidently rule out infection in the case of negative results and represents an important limitation, suggesting that dPCR cannot be used as the sole tool to rule out bloodstream infection [29,30,35]. These results are consistent with a recent meta-analysis that reported a pooled sensitivity of dPCR of 94% (95% CI, 85–98%) and a specificity of 87% (95% CI, 76–94%) when compared with blood culture [8]. A retrospective study of 115 septic patients reported similar results, with a sensitivity of 81.3% and a specificity of 56.6%, but with a high negative predictive value of 88.7% [36]. The variability in negative predictive values across studies may depend on the prevalence of infections in the study population, the pathogen panel included in the dPCR test, and the timing of sampling relative to antibiotic therapy initiation [8,29]. Likelihood ratio analysis further supports this interpretation, with a modest rule-in capability (LR+ 1.62) and limited rule-out performance (LR− 0.46). The negative likelihood ratio (LR−) of 0.46 and the low negative predictive value suggest that a negative dPCR result does not definitively rule out infection, particularly when the bacterial load is low or the pathogen is not included in the test panel [8,29,37]. The reduced turnaround time of dPCR (3–5 h) compared with blood culture (24–48 h or more) can enable more timely therapeutic escalation or de-escalation, improve clinical outcomes, and reduce the inappropriate use of broad-spectrum antibiotics [15,38,39]. Therefore, dPCR should be considered an add-on test rather than a replacement for blood culture, as recommended by guidelines from the Society of Critical Care Medicine and the Infectious Diseases Society of America [37]. An important advantage of dPCR is its ability to identify polymicrobial infections, which are often not detected by blood culture [35,36]. Recent studies have demonstrated that dPCR can detect mixed infections in 28.7% of cases at the time of ICU admission [30]. Furthermore, the absolute quantification of bacterial load provided by dPCR enables dynamic monitoring of infection progression and therapeutic response, with a correlation between microbial DNA copies and inflammatory markers such as procalcitonin and C-reactive protein [26,28,39,40]. The number of dPCR-negative samples raises questions about the analytical sensitivity of the method under conditions of low bacterial load or the presence of PCR inhibitors in blood [15,38]. The specificity of 53.8% also indicates the possibility of false-positive results, which may arise from contamination during sampling, detection of non-viable circulating microbial DNA, or colonization without invasive infection [15,29,36]. Recent studies have shown that the implementation of rigorous aseptic protocols can significantly reduce false positives, particularly for *Candida* spp. [36].

Blood culture was used as the conventional reference standard; however, the use of BC as the reference standard represents a major limitation, particularly in the context of sepsis. Indeed, blood culture sensitivity is known to be suboptimal, especially in patients receiving antimicrobial therapy, with a low pathogen burden, or with fungal infections [41]. Furthermore, culture-based methods may fail to identify pathogens in some cases of clinically suspected sepsis, highlighting the need for complementary diagnostic approaches [42]. Therefore, apparent dPCR-positive/BC-negative cases may not necessarily represent false positives. Rather than a definitive validation study, our work should be interpreted as an exploratory comparison between molecular detection and conventional microbiology. Future studies should employ composite clinical reference standards integrating microbiological, radiological and clinical data. To overcome this limitation, we applied a concordance-based framework integrating microbiological findings with the clinical context. Notably, a substantial proportion of discordant dPCR-positive cases was classified as clinically plausible. These included cases supported by microbiological evidence from other anatomical sites, polymicrobial infections, prior antimicrobial exposure, or fungal involvement. In such scenarios, dPCR likely detected circulating microbial DNA not captured by BC, consistent with previous studies showing that molecular assays can identify pathogens independently of viability and may retain diagnostic sensitivity despite ongoing antimicrobial therapy [43]. This aspect is particularly relevant in polymicrobial infections, which are increasingly recognized as common in critically ill patients but are often underdiagnosed by culture-based methods [44]. Similarly, the detection of fungal DNA by dPCR in cases with negative BC but positive cultures from other sites supports previous observations that molecular methods may enable earlier detection of invasive candidiasis, a condition associated with delayed diagnosis and high mortality [45]. Conversely, discordant cases without clinical support were interpreted as likely due to contamination, particularly when involving typical skin commensals such as coagulase-negative staphylococci. This finding underscores the importance of a careful interpretation of molecular results within the clinical context, as also emphasized in previous studies evaluating rapid molecular diagnostics for bloodstream infections [46]. Importantly, the concordance-based approach suggests that the apparent false-positive rate of dPCR may be overestimated when evaluated solely against BC. Similar observations have been reported in studies assessing molecular diagnostics, where so-called “false positives” often reflect true infections missed by conventional methods [47]. This highlights a broader methodological issue: traditional diagnostic accuracy metrics may not fully capture the clinical value of molecular assays in complex infectious diseases. Beyond diagnostic performance, dPCR demonstrated significant clinical added value in this study. The technique enabled rapid pathogen detection directly from whole blood, quantitative assessment of microbial load, and the identification of polymicrobial dynamics over time. These features are particularly relevant in the management of sepsis, where early and appropriate antimicrobial therapy is strongly associated with improved outcomes. Furthermore, the ability to detect antimicrobial resistance markers, such as carbapenemase genes (e.g., KPC, NDM, OXA-48), represents a key advantage over conventional methods, which often require prolonged incubation times. Rapid identification of resistance determinants has been shown to improve antimicrobial stewardship and optimize patient management in severe infections [48]. Beyond conventional diagnostic accuracy metrics, dPCR demonstrated a relevant clinical added value within the study cohort by enabling early pathogen detection, quantitative monitoring, and the identification of polymicrobial dynamics that were not consistently captured by blood culture. These features are particularly important in the context of sepsis, where timely and appropriate antimicrobial therapy is a key determinant of outcome [49]. The ability of dPCR to detect microbial DNA directly from whole blood, independently of pathogen viability, may explain its improved performance in patients receiving prior antibiotic therapy, a well-recognized limitation of culture-based diagnostics [41]. Longitudinal observations highlighted the capacity of dPCR to track changes in pathogen load over time and to detect persistent or emerging infections under antibiotic pressure. This is consistent with previous evidence showing that molecular methods can provide dynamic insights into infection evolution, potentially supporting real-time clinical decision-making [43]. In this context, the quantitative output of dPCR represents a significant advantage, as it may reflect microbial burden and help monitor the response to therapy, although further validation is required to define clinically relevant thresholds. Selected case-based analyses further illustrate the clinical utility of dPCR in complex infectious scenarios. Corynebacterium striatum (n = 2) and Citrobacter freundii (n = 1), isolated by blood culture, were not included in the dPCR panel and were therefore classified as false negatives in performance calculations; this may have underestimated dPCR sensitivity.

In one representative case, dPCR identified *Enterococcus faecalis* at low levels at admission and subsequently demonstrated a marked increase in bacterial load despite antibiotic therapy, together with the emergence of *Pseudomonas aeruginosa*. This finding suggests a polymicrobial infection associated with therapeutic failure and highlights the ability of dPCR to uncover infection complexity that may not be fully captured by blood culture. Polymicrobial bloodstream infections are increasingly recognized but remain underdiagnosed using conventional methods [50,51,52]. In another case, dPCR detected *Candida* spp. in peripheral blood with persistence over time in a patient with prior methicillin-resistant *Staphylococcus aureus* bacteremia and untreated urinary candidiasis. The temporal discordance between dPCR and blood culture findings suggests a complex infectious scenario potentially involving transient fungal dissemination. This observation is in line with previous studies demonstrating that molecular assays may enable earlier detection of invasive candidiasis compared with blood culture, which often shows limited sensitivity and delayed positivity [45]. A further example involved a patient in whom dPCR revealed a high load of *Escherichia coli* followed by a marked shift in the microbial profile after antibiotic therapy, with the emergence of additional Gram-negative pathogens and carbapenemase-producing organisms. This pattern is consistent with microbial selection under antibiotic pressure or the unmasking of previously undetected pathogens, highlighting the role of molecular diagnostics in identifying evolving antimicrobial resistance [48]. Additional cases demonstrated concordance between dPCR findings and microbiological results from other anatomical sites, supporting the ability of dPCR to detect bloodstream involvement secondary to localized infections. This is particularly relevant in immunocompromised or high-risk patients, where early dissemination may occur and timely detection is critical. Importantly, dPCR also enabled the early identification of multidrug-resistant organisms through the detection of resistance markers such as KPC, NDM, and OXA-48. Rapid recognition of such resistance determinants has been associated with improved antimicrobial stewardship and clinical outcomes, particularly in severe infections [48]. Furthermore, the detection of fungal DNA in patients with documented *Candida* infections at other sites, and in one case preceding blood culture positivity for *Candida parapsilosis*, supports the potential role of dPCR in the early diagnosis of invasive fungal infections. This is of clinical relevance given the high mortality associated with delayed antifungal therapy [45].

Taken together, these findings indicate that dPCR extends beyond simple pathogen detection by providing clinically actionable information, including the early identification of polymicrobial infections, monitoring of infection dynamics, detection of antimicrobial resistance-associated genes, and recognition of fungal involvement. Integration of dPCR with conventional microbiology and clinical data may therefore represent a promising approach for improving diagnostic accuracy and optimizing patient management in bloodstream infections.

## 4. Materials and Methods

### 4.1. Patient Cohort

The study included 37 samples from 27 patients admitted to Fondazione Policlinico Campus Bio-Medico di Roma between 16 December 2025, and 11 February 2026. Five patients contributed serial samples, resulting in a total of 37 paired samples from 27 patients. Diagnostic performance analyses were performed at the sample level, whereas serial samples were analyzed descriptively to illustrate longitudinal pathogen dynamics and treatment monitoring. Given the exploratory nature of this pilot study, no statistical adjustment for repeated measures was applied. Patients with suspected sepsis admitted to the following departments were included: cardiac surgery, general surgery, hematology, hepatology, geriatrics, immunohematology, internal medicine, clinical medicine, emergency medicine, medical pathology, the emergency room, intensive care, and urology. Information regarding the duration of antimicrobial exposure before sampling was not systematically available. Sepsis was defined according to Sepsis-3 criteria as suspected or documented infection associated with an increase in the SOFA score ≥ 2 points. Septic shock was defined according to Sepsis-3 criteria.

**Figure 1:**
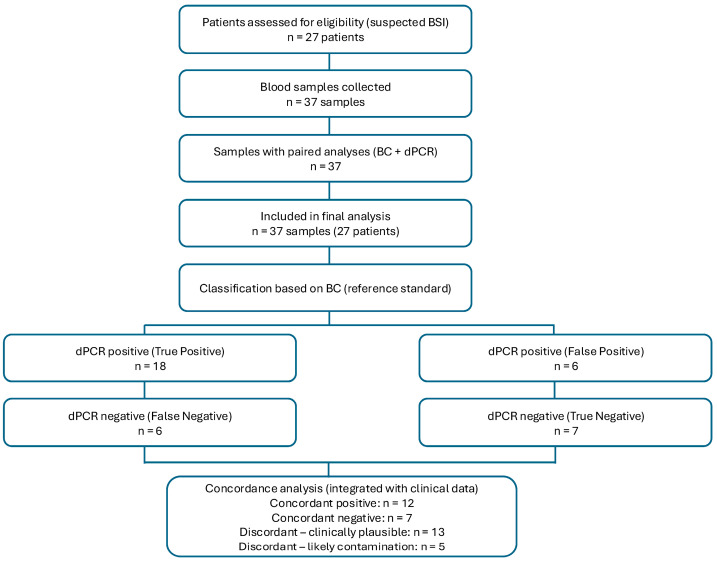
Flowchart of the samples/patients included.

This study analyzed venous blood samples received at our institution between 2025 and 2026 (Figure 1). All samples were collected using standard aseptic procedures and contained complete blood culture and dPCR results. dPCR and blood cultures were obtained simultaneously from paired blood samples. The study followed the Declaration of Helsinki and received approval from the Ethical Committee of the University Hospital Campus Bio-Medico of Rome (28.16TS Com Et CBM). Approval date 23 June 2016.

### 4.2. Blood Culture and Pathogen Detection

Whole blood samples were obtained for blood culture and molecular diagnosis when symptoms of infection were clinically suspected. According to the standard clinical procedure, sets of blood culture samples were collected for anaerobic and aerobic culture, with a venous blood collection volume of 10 mL/culture set. Then the samples were incubated at 37 °C in the BACTEC FX instrument (Becton Dickinson, Meylan, France). Positive samples were cultivated in selective agar media. Growing colonies were identified by MALDI-TOF (Bruker Daltonics GmbH, Bremen, Germany) [53,54,55,56,57,58,59,60]. Selective and nonselective media were used for microbiological cultures. Antimicrobial susceptibility testing was performed by Phoenix Instruments (BD, Beckton Dickinson, Franklin Lakes, NJ, USA), and MICs were interpreted according to Clinical and Laboratory Standards Institute (CLSI) M100-ED30 standards [61].

### 4.3. DNA Extraction and dPCR Assay

Approximately 5 mL of peripheral blood was collected by venipuncture into an EDTA anticoagulant tube and gently inverted 10 times (180° each time) to ensure adequate mixing.

Then, the plasma was immediately separated via centrifugation at 1200× *g* for 5 min at 4 °C. Plasma DNA was extracted using nucleic acid extraction or purification kits (Pilot Gene Technology, Hangzhou, China) [29] and the Auto-Pure10B Nucleic Acid Purification System according to the manufacturer’s instructions.

To detect the pathogens, a dPCR assay was performed using an automated droplet digital PCR system (Pilot Gene Technology, Hangzhou, China) according to the manufacturer’s instructions. Droplet production was performed using the equipment, and the PCR reaction was carried out according to the instructions. Once the process was completed, the cartridge was moved to the chip scanner for droplet analysis. Six fluorescence channels (FAM, VIC, ROX, CY5, CY5.5) were detected to identify the microorganisms in each panel, and data were analyzed using the GENEADP32 software version SW (V1.1.01.250312). According to the manufacturer’s validation, the pre-designed panel of the dPCR kit include primers/probes for *Pseudomonas aeruginosa*, *Escherichia coli*, *Klebsiella pneumoniae*, *Acinetobacter baumannii*, *Staphylococcus aureus*, *Candida* spp., *Enterococcus* spp., *Streptococcus* spp., *Stenotrophomonas maltophilia*, *Enterobacter cloacae*, *Proteus mirabilis*, Coagulase negative staphylococci, Serratia marcescens, as pathogens and KPC, mecA, OXA-48, NDM/IMP, vanA/vanB, as resistance genes. Molecular detection of resistance genes does not necessarily imply phenotypic resistance. According to the manufacturer’s technical documentation, the analytical performance of the MP02 dPCR assay includes a limit of detection (LoD) ranging from 20 to 50 copies/mL, depending on the target microorganism. The manufacturer reports an overall clinical sensitivity of ≥95% and clinical specificity of ≥90%, with target-specific sensitivities and specificities generally ranging from 95% to 100%. These analytical performance characteristics were established during the manufacturer’s validation studies and were not independently verified in the present study, which focused on evaluating the assay under routine clinical conditions. Information regarding the lower limit of quantification and inter-run reproducibility was not available in the manufacturer’s technical documentation and was therefore not assessed in the present study.

### 4.4. Data Analysis

Continuous variables that followed a normal distribution were expressed as mean ± standard deviation. Conversely, continuous variables that did not follow a normal distribution were expressed as the median and interquartile range (IQR). The diagnostic performance of digital PCR (dPCR) was evaluated using blood culture (BC) as the reference standard. Results were classified into true positives (TP), true negatives (TN), false positives (FP), and false negatives (FN) based on agreement between dPCR and BC at the sample level. A positive result was defined as the detection of at least one clinically relevant pathogen. In cases with multiple detected organisms, classification was based on the presence of at least one concordant pathogen between the two methods. Standard diagnostic accuracy metrics were calculated, including sensitivity (TP/[TP + FN]), specificity (TN/[TN + FP]), positive predictive value (PPV; TP/[TP + FP]), and negative predictive value (NPV; TN/[TN + FN]). In addition, positive and negative likelihood ratios (LR+ and LR−) were computed to assess the rule-in and rule-out performance of dPCR. Ninety-five percent confidence intervals (95% CIs) for sensitivity, specificity, positive predictive value, and negative predictive value were calculated using the Wilson score method. Given the known limitations of blood culture, particularly in the context of prior antimicrobial therapy, low microbial burden, and fungal infections, a secondary concordance-based analysis was performed to better interpret discordant results. Cases were categorized as concordant positive (both methods detecting the same pathogen), concordant negative (both methods negative), or discordant. Discordant cases were further subdivided into clinically plausible or likely due to contamination based on predefined criteria, including microbiological findings from other anatomical sites, consistency across multiple samples or time points, the presence of a polymicrobial infection, and the clinical context. Cases involving typical skin commensals (e.g., coagulase-negative staphylococci) detected in a single sample without supporting clinical evidence were classified as a likely contamination. Conversely, dPCR-positive/BC-negative cases supported by additional microbiological or clinical data (e.g., infections at other anatomical sites, antifungal or antibiotic exposure, or the detection of antimicrobial resistance-associated gene markers) were considered clinically plausible. All analyses were descriptive due to the pilot nature of the study. Data were summarized as counts and percentages, and no formal hypothesis testing was performed.

### 4.5. Limitations

Several limitations of the present study should be acknowledged. First, this was a single-center pilot study with a relatively small sample size, which limits the generalizability of the findings and precludes definitive conclusions regarding diagnostic accuracy. In addition, five patients contributed serial samples. Although these longitudinal observations provided valuable insights into pathogen dynamics and treatment monitoring, repeated measurements may have affected the assumption of independence between observations and potentially influenced estimates of diagnostic performance. Second, blood culture was used as the reference standard. However, the sensitivity of blood culture is known to be suboptimal, particularly in patients receiving antimicrobial therapy, in cases of low microbial burden, or in fungal infections. Consequently, some dPCR-positive/blood culture-negative cases classified as false positives may represent clinically relevant infections not captured by conventional microbiology. Therefore, the reported sensitivity and specificity values should be interpreted with caution and viewed as exploratory rather than definitive measures of diagnostic accuracy. Future studies should consider composite clinical reference standards integrating microbiological, radiological, and clinical findings. The dPCR assay was based on a predefined panel of microorganisms and resistance-associated genes. Accordingly, pathogens not included in the panel could not be detected, potentially leading to an underestimation of assay sensitivity. Moreover, the detection of resistance genes does not imply phenotypic antimicrobial resistance and requires confirmation by conventional susceptibility testing. Furthermore, information regarding the duration and timing of antimicrobial therapy before sample collection was not systematically available for all patients. Since molecular methods are less dependent on microbial viability than culture-based techniques, previous antibiotic exposure may have influenced the degree of concordance between dPCR and blood culture. Finally, the observational nature of the study and the absence of clinical outcome analyses preclude the assessment of whether the implementation of dPCR translates into improved patient outcomes, reduced mortality, shorter hospital stays, or enhanced antimicrobial stewardship. Larger prospective multicenter studies are warranted to validate these findings and to better define the role of dPCR as a complementary tool in the diagnostic pathway of bloodstream infections.

## 5. Conclusions

dPCR represents a promising tool for improving the management of bloodstream infections, particularly in critical settings where diagnostic speed is essential. Integration of dPCR with traditional blood culture can increase the overall pathogen detection rate and support antimicrobial stewardship programs, enabling early targeted therapy and reducing the use of broad-spectrum antibiotics. However, prospective implementation studies with clinical endpoints (mortality, length of stay, healthcare costs) are needed to assess the real impact of dPCR on patient outcomes.

In conclusion, the results of this study confirm that dPCR offers significant advantages in terms of speed, detection spectrum, and the ability to identify polymicrobial infections, while presenting limitations in negative predictive value that restrict its use as a standalone test. The optimal approach consists of the complementary use of dPCR and blood culture, leveraging the strengths of both methods to improve the diagnosis and management of bloodstream infections.

## Figures and Tables

**Table 1:** General clinical characteristics of patients.

Variables	Mean (SD)	Median (IQR)
Age in years, median	67 (13)	69 (56–78)
Sex category, number male (%)	14 (52%)	-
WBC, 10^3^/µL	7 (6)	5.3 (2.7–10.5)
PCT, ng/mL	2 (4)	0.5 (0.2–2.1)
CRP, mg/L	9 (7)	8.7 (2.6–11.9)

**Table 2:** Comparison of species detected by dPCR and isolated by blood culture.

Species	dPCR	Blood Colture
*Enterococcus*	6	5
*P. aeurigonosa*	4	2
*Streptococcus*	2	0
*A. baumannii*	2	1
Coagulase-negative staphylococcus	5	8
*E. coli*	2	4
*P. mirabilis*	2	0
*K. pneumoniae*	5	2
*S. aureus*	3	5
*C. striatum*	0	2
*C. freundi*	0	1
*Candida*	6	2
Negative	12	8

**Table 3:** Performance between dPCR and blood culture.

	BC Positive	BC Negative
**dPCR Positive**	18 (TP)	6 (FP)
**dPCR Negative**	6 (FN)	7 (TN)

**Table 4:** Concordance analysis between dPCR and blood culture with clinical interpretation.

Category	n (%)	Representative Findings	Clinical Interpretation
Concordant positive	12 (~32%)	*E. faecalis*, *E. coli*, *P. aeruginosa*, *A. baumannii*, *K. pneumoniae*, *S. aureus*, *Candida* spp.	Confirms bloodstream infection; strong agreement between methods
Concordant negative	7 (~19%)	No pathogen detected	True negative cases; no evidence of bloodstream infection
Discordant—clinically plausible	13 (~35%)	*Candida* spp. with urinary source; MDR Gram-negatives (KPC, NDM, OXA-48); polymicrobial profiles; prior antibiotic exposure	Likely true infections not captured by BC; reflects higher sensitivity of dPCR and ability to detect polymicrobial/fungal involvement
Discordant—likely contamination	5 (~14%)	Coagulase-negative staphylococci (*S. epidermidis*, *S. caprae*), single-timepoint *E. coli*	Likely false-positive BC or clinically irrelevant findings

**Table 5:** Clinical scenarios where dPCR provides added value in bloodstream infection management.

Clinical Scenario	Rappresentative Case	Key dPCR Finding	Added Clinical Value	Implication for Practice
Persistent infection under therapy	Case 1	Increase in *E. faecalis* load despite antibiotics; detection of *P. aeruginosa*	Early identification of treatment failure and polymicrobial infection	Supports timely escalation and therapy adjustment
Bacterial–fungal co-infection with temporal discordance	Case 2	Detection of *Candida* spp. prior to and independent from BC; coexistence with MRSA	Early recognition of fungal involvement and complex infection dynamics	Promotes earlier antifungal consideration and integrated interpretation
Microbial shift under antibiotic pressure	Case 3	Emergence of multiple Gram-negative pathogens and KPC after meropenem	Detection of evolving resistance and polymicrobial ecology	Enables real-time adaptation of antimicrobial strategy
Local-to-systemic infection link	Case 4	Detection of *Enterococcus* spp. consistent with wound culture (VRE)	Identification of bloodstream involvement from localized infection	Supports source control and targeted therapy
Complex polymicrobial infection in frail patients	Case 5	Simultaneous detection of *Streptococcus* spp. and *S. aureus*	Improved detection of polymicrobial or evolving infections	Enhances diagnostic accuracy in high-risk populations
Disseminated MDR infection (ICU setting)	Case 6	Detection of *A. baumannii*, *K. pneumoniae* and KPC	Early identification of multidrug-resistant pathogens	Critical for rapid antimicrobial stewardship and infection control
Fungal detection from non-blood source	Case 7	Detection of *Candida* spp. with concurrent urinary infection	Identification of possible fungal dissemination or DNAemia	Supports early suspicion of invasive candidiasis
Combined AMR and fungal detection	Case 8	Detection of *K. pneumoniae* (NDM, OXA-48) and *Candida* spp.	Simultaneous identification of resistance profile and fungal component	Enables precision medicine approach in severe infections

## Data Availability

The original contributions presented in this study are included in the article and Supplementary Materials. Further inquiries can be directed to the corresponding author.

## Supplementary Materials

The following supporting information can be downloaded at: https://www.mdpi.com/article/10.3390/ijms27156849/s1.
