# Supplementary material for: Fast Microbiology: A Pilot Study Comparing Culture-Independent Digital PCR and Blood Culture for Pathogen Detection in Sepsis
Source: Int J Mol Sci. 2026 Jul 30;27(15):6849. doi: 10.3390/ijms27156849 (PMC13466399; doi:10.3390/ijms27156849)
Supplement: Supplementary file 1 [file ijms-27-06849-s001.zip › ijms-4372560-supplementary.pdf]

Fast Microbiology: a pilot study comparing culture-independent digital PCR and blood culture for pathogen detection in sepsis

Maria Vittoria Ristori[1,2,3], Tiziana Marfoli[2], Marina De Cesaris[2], Sara Elsa Aita[4], Raffaella Rosy Vescio[2], Francesco Travaglino[5], Silvia Spoto[6], Raffaele Antonelli Incalzi[7,8], and Silvia Angeletti[2,3]*

Supplementary material

Table S1. Copies/ul of the results obtained with dPCR

Sample ID	dPCR results	Copies/ul
1	Negative	0
13	Negative	0
14	Negative	0
2	Enterococcus	2,07
6	Enterococcus	158
	P. Aeurigonosa	50,0
4	Candida	76,86
5	Streptococcus	3,46
	Enterococcus	1,15
7	Negative	0
8	Negative	0
9	Negative	0
10	Candida	2,77
19	Candida	3,11
11	P. Aeruginosa	2,37
12	P. Aeruginosa	2,77
15	A.Baumannii	79,45
16	Staphylococcus coagulase-negative	7208,53
	mecA	9000,97
17	Negative	0

18	Negative	0
24	Negative	0
20	Candida	35,70
	Enterococcus	1,13
	Staphylococcus coagulase-negative	5,36
21	Staphylococcus coagulase-negative	5,37
	mecA	7,63
22	E.Coli	612,53
	Proetus Mirabilis	3,2
23	E.Coli	83,18
	P. Mirabilis	0,92
	P. Aeruginosa	812,77
	K.Pneumoniae	79,23
	KPC	22,8
25	Enterococcus	0,78
	Staphylococcus coagulase-negative	1,14
26	K.Pneumoniae	0,83
	Enterococcus	1,05
	KPC	0,76
27	Streptococcus	4,74
	S.Aureus	10,09
28	A.Baumannii	1,03
	K.Pneumoniae	1,12
	KPC	0,53
29	Candida	3,25
30	K. Pneumoniae	1149
	NDM	265,87
	OXA 48	265,37
	Candida	589,81
31	S. aureus	0,96
32	Staphylococcus coagulase-negative	5,19
	mecA	10,32

33	K. Pneumoniae	1,33
	KPC	0,72
34	Negative	0
35	S. aureus	21,88
36	Negative	0
37	Negative	0
3	Negative	0

Table S2. Comparison table of the times required by each method

Test	Time
dPCR	3–5 h
BC positivity	18–48 h
Identification + AST	48–96 h
